# Supplementary material for: Universal superconducting precursor in three classes of unconventional superconductors
Source: Nat Commun. 2019 Jun 21;10:2729. doi: 10.1038/s41467-019-10635-w (PMC6588566; doi:10.1038/s41467-019-10635-w)
Supplement: Supplementary file 1 — Supplementary Information [file 41467_2019_10635_MOESM1_ESM.pdf]

Supplementary Materials

Universal superconducting precursor in three classes of unconventional superconductors

Pelc et al.

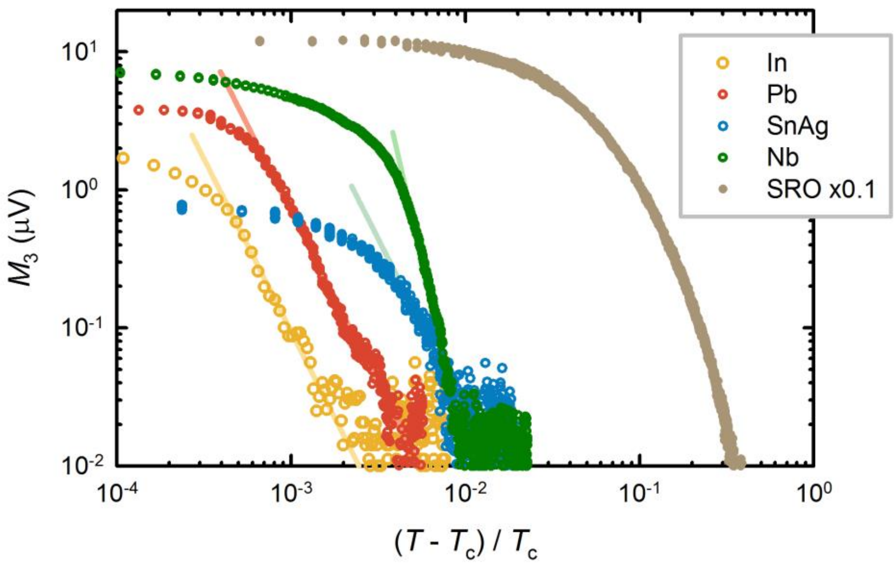

Supplementary Figure 1 | Nonlinear magnetization of four conventional superconductors. The nonlinear response of the conventional superconductors indium, lead, tin-silver alloy and a niobium single crystal is compared to that for strontium ruthenate on a relative, reduced temperature scale. In, Pb and SnAg follow the mean-field Ginzburg-Landau prediction[1] of a power-law tail with exponent -5/2, whereas the exponent for niobium is somewhat larger at -3.6. The SRO data, in contrast, do not show power-law behaviour and extend to much higher reduced temperatures.

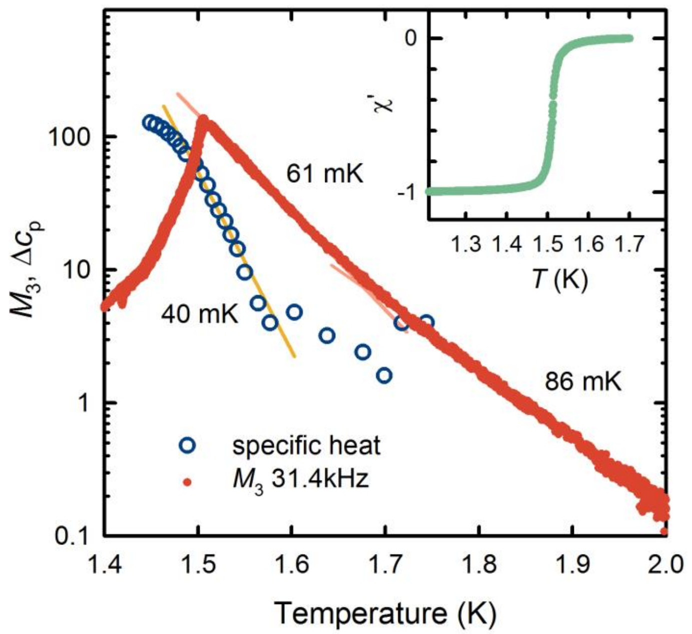

Supplementary Figure 2 | SRO linear susceptibility and specific heat. Nonlinear response (full circles) compared to electronic specific heat data (empty circles, from ref. 4) with the constant normal-state specific heat contribution subtracted. The specific heat data are consistent with an exponential decay, over roughly one decade in the given temperature range above T_c (that is limited due to signal-to-noise). The slope of the specific heat data (~ 40 mK) is steeper than the slope of the nonlinear response. The inset shows the linear susceptibility of the T_c = 1.51 K SRO sample and demonstrates a sharp superconducting transition.

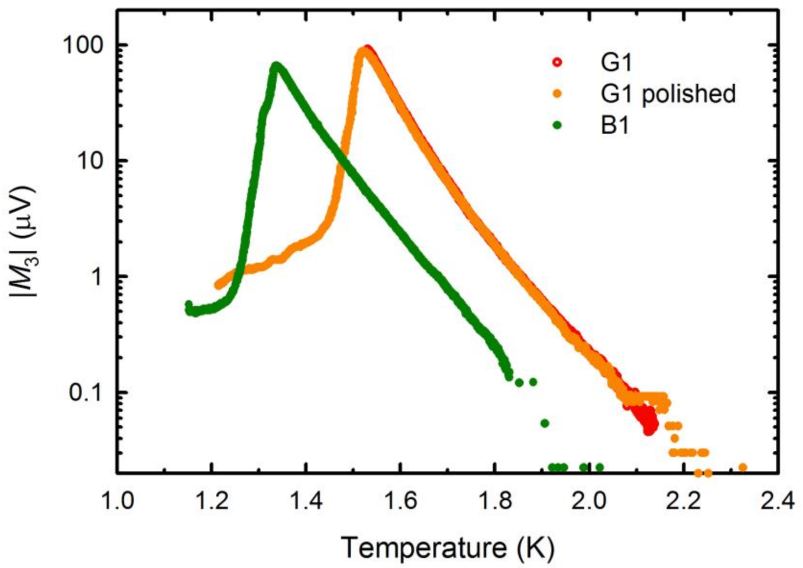

Supplementary Figure 3 | Comparison of different Sr_2RuO_4 samples. Measurements of third-order response for two SRO samples with T_c of 1.51 K (sample G1) and 1.33 K (sample B1) demonstrate closely similar behaviour above their respective T_c. A comparison of measurements of the G1 crystal before and after additional polishing is shown as well. The polishing was done with the intent to remove some inclusion-containing material, yet no difference in the response is discerned.

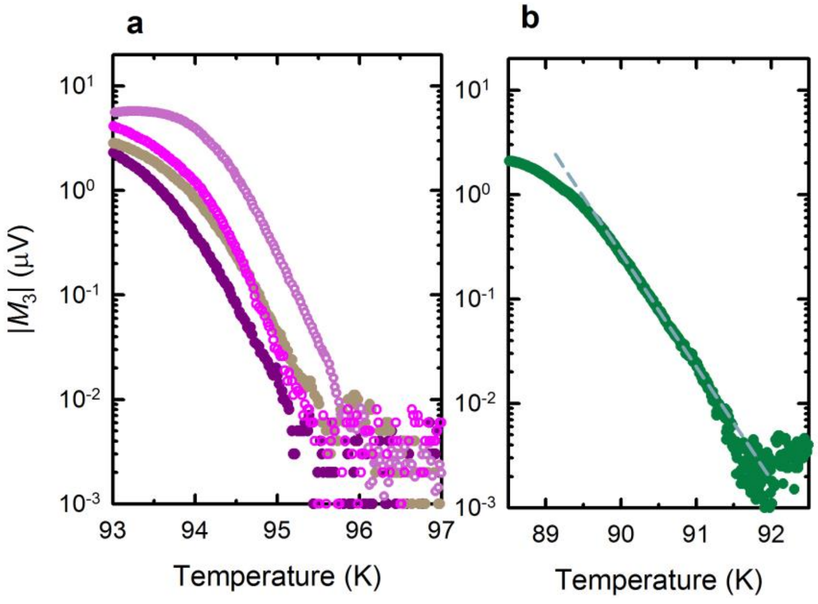

Supplementary Figure 4 | Comparison of several Hg1201 samples. **a**, Nonlinear magnetization measurements for four samples from two different growth batches show similar exponential tails. **b,** A sample with slightly smaller T_c exhibits the same behaviour. The overlaid dashed line demonstrates consistency with simple exponential decay over almost two decades. The slight bump at the high-temperature end likely results from surface superconductivity.

Supplementary Notes

Supplementary Note 1: Conventional superconductors. The study of superconducting fluctuations in conventional systems is hampered by the extremely small temperature range above T_c where such effects can be observed in nonlinear response. In order to study nonlinear effects in conventional superconductors, we chose four different materials: In (T_c = 3.4 K, type I), Nb (T_c = 9.31 K, type II), Pb (T_c = 7.2 K, type I) and a SnAg alloy (T_c = 3.67 K, type I). In, Pb and SnAg were polycrystalline samples, while Nb was a single crystal (see also Methods). The measured third-order signals are shown in Supplementary Figure 1 on a relative temperature scale, $\epsilon = (T - T_c)/T_c$, and compared to SRO (from Figure 1), which was chosen because of the very good signal-to-noise ratio. Clearly, the response of conventional superconductors quickly decreases above T_c and displays a power-law tail over at least 1-2 orders of magnitude in M_3. Mean-field Ginzburg-Landau theory predicts that the third-order magnetization should decrease as $\epsilon^{-5/2}$ [1] for small applied magnetic fields and at relatively large ϵ; this prediction is essentially borne out by these data. Nb is an exception, as it shows power-law behaviour with a somewhat larger exponent of -3.6, but clearly the behaviour of conventional systems qualitatively differs from that of the perovskite-based superconductors.

Supplementary Note 2: Ru inclusions in SRO. Strontium ruthenate single crystals often contain inclusions of ruthenium metal due to the growth conditions that require an excess of Ru. It is known that T_c is locally enhanced around the inclusions[2] (within the SRO, not the Ru metal), most likely due to the induced local stress[3]. Crystals with the highest achievable T_c ~ 1.5 K can still contain a non-negligible concentration of Ru inclusions, i.e., their number is unrelated to the level of point defects. Due to the strong local enhancement of T_c, it is possible that the material around inclusions contributes to the nonlinear susceptibility far above the bulk T_c. As a result of the high sensitivity of our technique, it can detect inclusion-related signals at the level of 10^{-3} Ru volume fraction or lower. However, it seems unlikely that the inclusions would qualitatively change the nonlinear response in the entire range from T_c to ~ 2 K, especially close to T_c (the transition is sharp and well defined in the linear susceptibility; see Supplementary Figure 2 inset). Conceivably, the high-temperature tail of the response is influenced by the inclusions, which might explain the apparent change in slope of the exponential dependence (Supplementary Figure 2). Furthermore, in Supplementary Figure 2 we compare the nonlinear susceptibility with

specific heat measurements[4], where the behaviour above T_c seems consistent with an exponential decay (although the temperature range for which data are available is somewhat narrow). The extracted characteristic temperature is about 50% smaller than the equivalent slope of the nonlinear susceptibility close to T_c, and approximately two times smaller than the high-temperature slope. Importantly, the temperature dependence is qualitatively similar.

In an effort to further investigate the possible influence of inclusions, we have performed nonlinear response measurements on a SRO sample from a different growth, and with a lower T_c of 1.33 K (Supplementary Figure 3). The slopes of the exponential tails are virtually the same for the two samples, despite differences in T_c and crystal growth conditions, which suggests that the observed behaviour is at best weakly influenced by Ru inclusions. We further compare the results to measurements on the same T_c = 1.51 K sample after additional polishing (in an attempt to remove some inclusion-containing material). Again, no significant difference is observed.

Supplementary Note 3: Additional Hg1201 measurements. In order to investigate the typical sample-to-sample variability in Hg1201, we performed measurements on several crystals from different growth batches with transition temperatures close to the optimal value (Supplementary Figure 4). All samples show similar behaviour. Notably, in Hg1201 the carrier doping process involves oxygen diffusion, so it is not uncommon to see additional features in the response above T_c; a possible example of this is the slight bump in Supplementary Figure 4b around 91 K. The third-order response is extremely sensitive to such effects.

Supplementary References

[1] Tsuzuki, T. & Koyanagi, M. Nonlinear diamagnetism due to the fluctuation superconductivity. *Phys. Lett. A* **30**, 545-546 (1969).

[2] Maeno, Y. et al. Enhancement of superconductivity of Sr_2RuO_4 to 3 K by embedded metallic microdomains. *Phys. Rev. Lett.* **81**, 3765-3768 (1998).

[3] Steppke, A. et al., Strong peak in T_c of Sr_2RuO_4 under uniaxial pressure. *Science* **335**, eaaf9398 (2017).

[4] Nishizaki, S., Maeno, Y. & Mao, Z., Effect of impurities on the specific heat of the spin-triplet superconductor Sr_2RuO_4. *J. Low. Temp. Phys.* **117**, 1581-1585 (1999).
